# Loneliness in caregivers of people with chronic illnesses: conceptual analysis according to Walker and Avant

**DOI:** 10.1590/0034-7167-2024-0651

**Published:** 2026-02-23

**Authors:** Claudia Patricia Cantillo Medina, Marcos Venícios de Oliveira Lopes, Viviane Martins Silva, Reinaldo Gutierrez Barreiro, Janaina Fonsecca Victor Coutinho, Claudia Andrea Ramírez-Perdomo

**Affiliations:** 1 Universidad Surcolombiana. Neiva, Huila, Colombia.; 2 Universidad Federal de Ceará. Fortaleza, Ceará, Brazil.

**Keywords:** Caregivers, Chronic Disease, Concept Formation, Nursing Research, Loneliness

## Abstract

**Objectives:**

to analyze the concept of loneliness in caregivers of people with chronic noncommunicable diseases.

**Methods:**

concept analysis proposed by Walker and Avant. A quantitative scoping review was incorporated to identify the available scientific literature, based on the theoretical framework of the Joanna Briggs Institute. It included 30 articles from the Scopus and PubMed databases, published between 2015 and 2024 in Spanish, English, and Portuguese, with a score greater than or equal to 8/10 according to the Critical Appraisal Skills Programme Español.

**Results:**

the background, attributes, and consequences identified in the conceptual analysis of “loneliness” in caregivers allowed us to describe the effects of caregiving on physical, emotional, social, and spiritual well-being.

**Conclusions:**

revealed the complexity and multidimensional trajectory of the phenomenon of loneliness, which allows for the proposal of policies aimed at its prevention and contributes to the well-being of the population.

## INTRODUCTION

Loneliness is a universal phenomenon experienced by all individuals throughout their lives. Its increased prevalence during the Covid-19 outbreak has led to its definition as a global public health problem^(1)^. At the same time, there has been a global increase in chronic noncommunicable diseases (CNCDs), which cause reduced functionality and dependence on care; conditions that specifically affect individuals, families, and caregivers. For this reason, they are considered important causes of suffering and disability, with a significant economic and social burden^(12)^.

In Europe, different countries in the north and west reported a prevalence of loneliness among caregivers of between 10% and 20%, reaching approximately 25% during the first five months of the Covid-19 outbreak. In Eastern European countries, the prevalence rates observed were higher, ranging from 30% to 55%^(1)^. In the United Kingdom, the prevalence was 21%; in 28 countries in Africa, Asia, and the Americas, low- and middle-income countries, significant upward trends were observed^(1)^.

Caregivers are usually family members, friends, or neighbors who provide unpaid assistance to others with physical, psychological, or cognitive limitations, and often find themselves unexpectedly committed to their role. They must support the person being cared for in their daily life, administer medication, use equipment, organize follow-up appointments, and decide and resolve situations related to well-being and health, often without adequate preparation for the complexity of their loved one’s health and life in the home context^(3)^.

Due to the demand for attention and the time invested in performance, caregiving is considered a chronic stressor at the psychological level and is associated with the presence of anxiety, depression, overload, and loneliness symptoms^(4-6)^. The literature shows an association between objective and subjective stress and daily care, contributing directly to the emergence of feelings of loneliness, related to the greater manifestation of depressive symptoms^(7)^. Caregivers often struggle with losses, deprivations, and distancing from their social circles, reduced personal space, and interpersonal relationships^(8)^, circumstances that intensify feelings of loneliness^(9)^.

Among caregivers with access to social support, there is better fulfillment of the role and self-care activities, greater empowerment, and ability to use available resources; in addition, feelings of loneliness and psychological problems are reduced^(10)^. The perception of loneliness is defined as a painful experience that occurs when social networks are deficient; people suffer loneliness when they perceive a deficit in their relationships, either because they have fewer than desired or because they do not provide the intimacy they need^(11)^.

The loneliness perceived by caregivers is described as the difference between expectations about interpersonal relationships with others, in reference to the quality or veracity of the connections felt, in contrast to their reality^(11)^. Its chronic presentation can be a cause of premature mortality and chronic diseases^(12)^. From a biopsychosocial perspective, the availability of social relationships is a tool for promoting health and well-being^(13)^. Understanding the concept of loneliness in caregivers of people with NCDs is an important step toward closing the conceptual gap regarding this reality and can serve as a basis for proposing policies and implementing intervention strategies aimed at informal caregivers in the prevention of loneliness.

## OBJECTIVES

To analyze the concept of “loneliness” in caregivers of people with NCDs.

## METHODS

The study followed a theoretical approach using Walker & Avant’s concept analysis method, known for its rigor and precision, which consists of eight steps^(14)^:

1. Select the concept: this is determined to clarify the definition of the phenomenon under study, in order to avoid inaccurate use in nursing theory and research^(14)^.
2. Determine the purpose of the analysis: this represents the exploration of the concept in a broad and systematic way, based on understanding the phenomenon as a basis for generating policies, strategies, and interventions that guide care practice^(14)^.
3. Identify the uses of the concept: this establishes the ways in which the concept is employed and reveals the descriptive attributes of the phenomenon^(14)^.
4. Determine the defining characteristics or attributes of the concept: each concept contains more than one defining attribute, which are repeated repeatedly and are key in differentiating it from others; it is necessary to determine which attribute is most appropriate to describe it^(14)^.
5. Identify the antecedents of the concept: understand the possible circumstances, contexts, or events that occur before the concept^(14)^.
6. Consequences of the concept: these are events or results that may occur after the concept or as a product of the concept^(14)^.
7. Define empirical references: these show the methods for measuring concepts and the extent to which the definition can be useful for measuring and validating the concept, as well as for producing research tools^(14)^.
8. Identify a model case and identify related cases: the model or case is a pure example of the concept under study and must present all the defining attributes^(14)^; the related case presents an example of the concept under study that represents some of the defining attributes^(14)^.

To obtain data and identify the elements of concept analysis, a quantitative scoping review was conducted, based on the theoretical framework of the Joanna Briggs Institute (JBI)^(15)^; The recommendation of the protocol established by the Preferred Reporting Items for Systematic Reviews and Meta-Analyses for Scoping Reviews (PRISMA-ScR)^(15)^ was adopted. The review process followed the guidelines proposed by Arksey and O’Malley^(16)^, used to identify the key concepts that underpin a field of research, as well as to clarify working definitions and/or the conceptual boundaries of a topic. This involved identifying key sources of evidence, synthesizing and mapping the main characteristics of the included studies, such as the research designs used, the target populations, the interventions or exposures evaluated, and the reported results^(16)^.

The research question that guided the review used the PCC strategy and, in this sense, was structured as follows: (P): Family caregivers of people with chronic noncommunicable diseases; (C): Concept of loneliness; (C): Worldwide.

Thus, the guiding question was: What are the uses, attributes, antecedents, consequences, and empirical references of the concept of loneliness in caregivers of people with CNCDs?

The review and verification process included five steps:

Step 1: Database search.

The review protocol was developed by the researchers in the Scopus and PubMed databases from July to September 2024; the Boolean operators “AND” and “OR” were used, which related the following DeCS-MeSH search terms, combining the keywords: Caregivers; Chronic Disease; Concept Formation; Nursing research; Loneliness; the equation was adjusted in each of the databases (Chart 1).

**Chart 1:** Search terms with DeCS and MeSH descriptors

Databases	Search strategy in English	Results
Scopus	“Chronic Disease” OR “Noncommunicable Diseases” AND “Caregivers” OR “Informal Caregivers” OR “Familiar Caregivers” AND “Loneliness” OR “Social Alienation”	20
PubMed	((((“Chronic Disease”[Mesh]) OR “Noncommunicable Diseases”[Mesh]) AND “Caregivers”[Mesh]) AND “Loneliness”[Mesh]) OR “Social Alienation”[Mesh]	10

Step 2: Inclusion criteria.

The articles selected were those written in Spanish, English, and Portuguese, published between 2015 and 2024, quantitative and qualitative studies published in indexed journals; they included experimental, descriptive, clinical trials, pilot studies, and qualitative studies. The articles were compiled in Microsoft Excel® in a matrix in which categories of information were defined; the methodological quality of the article was criticized using the Critical Appraisal Skills Programme Español (CASPe)^(17)^, whose objective is to provide the necessary skills for the “critical reading of clinical evidence”; Articles that obtained a score greater than or equal to 8/10 were selected. Grey literature, letters, comments, expert opinions, and editorials were excluded.

Step 3: Data analysis.

Each study was analyzed individually, and relevant data were incorporated into a descriptive chart with information on the authors, year of publication, location, definition of the concept of loneliness in caregivers of people with NCDs, use, defining characteristics or attributes, background, consequences, empirical references, results, and main conclusions.

Step 4: Full-text verification.

A full-text reading was performed to verify the contribution of each selected study. Relevant data were extracted, including study design, participant characteristics, identified factors, main results, and conclusions. These were then systematically summarized.

Step 5: Synthesis and presentation of results

For a better understanding of the phenomenon, the themes and patterns extracted from the articles that prevailed in the studies were identified, as well as patterns, trends, and gaps in the literature. Analytical categories congruent with the area of interest were assigned and grouped thematically^(16)^. This study did not require approval from the Ethics and Bioethics Committee as it did not involve human beings; copyright principles were respected in the use of references from the scientific material analyzed.

## RESULTS

**Figure 1:**
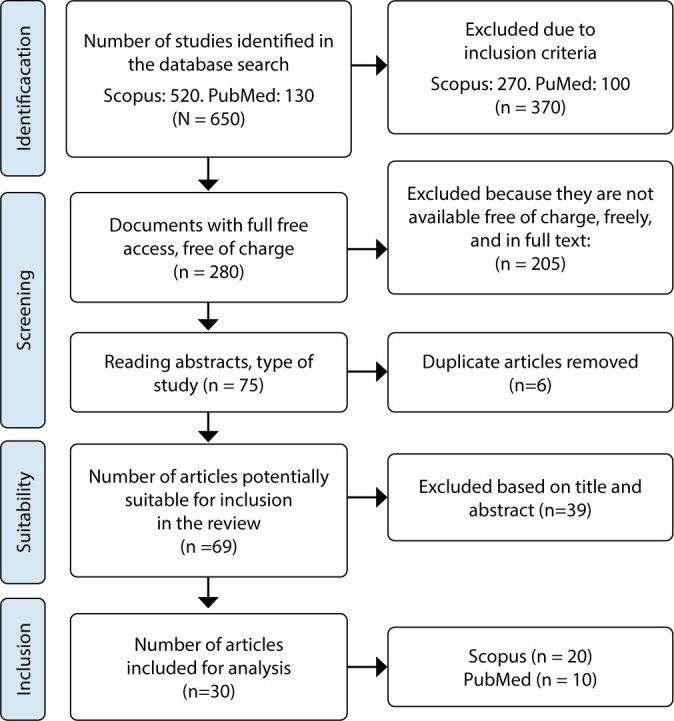
PRISMA-ScR flow diagram of the strategy for searching and selecting articles in databases, 2024

A total of 650 potentially eligible studies were identified. Based on predetermined selection criteria, 370 documents were eliminated, 205 because they were not available free of charge and in full text, 6 duplicate articles, and 39 after reviewing titles and abstracts. The references of the articles and those considered relevant to the topic of caregiver loneliness were reviewed manually. Thirty articles were selected for full reading and analysis, as shown in Figure 1.

The results of the scope review were consolidated by describing the general characteristics of the selected studies (Chart 2). The sample consisted of 30 studies from the international and national literature published in indexed journals between 2015 and 2024 (Chart 2). The articles included were published in the following countries: United Kingdom (7), Colombia (4), United States (3), Norway, Mexico, Cuba (2), Germany, Spain, France, Netherlands, Turkey, Iran, China, Singapore, Chile, and Brazil (1).

**Chart 2:** Summary of articles analyzed in the scoping review on the analysis of the concept of loneliness in caregivers of people with NCDs, (N=30), 2024

No	Author	Title or reference	Design/ participants	Sample/objective	Country / Year	Database	CASPe
1	SerçekuÅ P. Becoming a family caregiver of a patient living with cancer^(3)^	Becoming a family caregiver of a patient living with cancer.	Autoethnography	1	Turkey (2020)	PUBMED	CASPe 8/10
2	Victor CR, Rippon I, Quinn C, Nelis SM, Martyr A, Hart N, et al.^(8)^	The prevalence and predictors of loneliness in caregivers of people with dementia: findings from the IDEAL programme	Cohort study Improving the experience of Dementia and Enhancing Active Life (IDEAL)	1,283 family caregivers of people with mild to moderate dementia living in Great Britain.	Great Britain (2021)	PUBMED	CASPe 10/10
3	Blanco-Martínez L, Simón-Álvarez A, Sánchez-Fernández A^(18)^	Caracterización biopsicosocial de los cuidadores informales de pacientes con enfermedad crónica o terminal	Descriptive cross-sectional study	87 caregivers of people with chronic illness	Cuba (2016)	SCOPUS	CASPe 9/10
4	Hejazi S, Manzour R, Shahsavari A, Ghasemi S, Roshan-Nejad M^(19)^	Experiences of family caregivers of individuals undergoing hemodialysis in Iran about caring during the COVID-19 pandemic: a qualitative study	Qualitative, using inductive qualitative content analysis.	17 family caregivers of people undergoing hemodialysis in Bojnurd, Iran	Iran (2024)	SCOPUS	CASPe 9/10
5	Hui Y, Wang Y, Dongxia Xiao L, Liu L^(20)^	Prevalencia y factores asociados con síntomas psicológicos negativos entre viudas mayores que viven solas en una muestra remota de China: un estudio transversal.	Cross-sectional study	271 widows	China (2023)	SCOPUS	CASPe 9/10
6	Holton E, Bernadette Boyle N, Simons R, Warters A, O'Philbin L, Lawlor B, et al.^(21)^	Freedom and loneliness: dementia caregiver experiences of the nursing home transition	Qualitative study, semi-structured interviews were conducted.	11 caregivers	Ireland (2023)	PUBMED	CASPe 8/10
7	Carrillo Cervantes AL, Medina Fernández IA, Medina Fernández JA, Chaparro Díaz L, Carreño Moreno SP, Cortez González LC^(22)^	Soledad, ansiedad, depresión y adopción del rol de cuidador de adultos mayores con enfermedad crónica durante la covid-19.	Predictive correlational design.	157 caregivers	Mexico (2022)	SCOPUS	CASPe 9/10
8	Mares-Rico KF, Cardona-Ramírez VL, Franco-Álvarez DM, Medina-Fernández IA, Medina-Fernández JA, Carrillo-Cervantes AL^(23)^	Ansiedad, depresión y soledad en cuidadores de personas con enfermedad crónica.	Correlational descriptive design.	178 family caregivers	Mexico (2022)	SCOPUS	CASPe 9/10
9	Miller K; Van Houtven C, Smith V, Hoff Lindquist J, Gray K, Richardson C, et al.^(24)^	Los cuidadores familiares de veteranos experimentan niveles clínicamente significativos de angustia antes y durante la pandemia: Implicaciones para los servicios de apoyo a los cuidadores	Pre- and post-design and longitudinal data.	903 caregivers	United States (2022)	PUBMED	CASPe 10/10
10	Carreño-Moreno S, Pacheco-López M, Arias-Rojas M^(25)^	Role adoption, anxiety, depression and loneliness in family caregivers of patients with chronic diseases	Descriptive, exploratory, and cross-sectional study.	960 family caregivers	Colombia (2024)	SCOPUS	CASPe 10/10
11	Valenzuela M, Slachevsky A, Thumala D, Pinto A, Olavarría L, Lema J, et al.^(26)^	Soledad en cuidadores informales de personas con demencia durante la pandemia de Covid-19	Cross-sectional quantitative study.	195 caregivers	Chile (2023)	SCOPUS	CASPe 9/10
12	Saadi JP, Carr E, Fleischmann M, Murray E, Head J, Steptoe A, et al.^(27)^	The role of loneliness in the development of depressive symptoms among partnered dementia caregivers: Evidence from the English Longitudinal Study of Aging	English Longitudinal Study on Aging (ELSA).	10,813 people who had provided care to their partner between waves of the study (ELSA)	England (2021)	SCOPUS	CASPe 9/10
13	Parada-Rico DA, Carreño-Moreno S, Chaparro-Díaz OL^(28)^	Soledad, ansiedad y depresión en la adopción del rol de cuidador familiar del paciente crónico	Cross-sectional quantitative analytical research	120 Caregivers	Colombia (2023)	SCOPUS	CASPe 9/10
14	Koopman E, Heemskerk M, van derBeek AJ, Coenen P^(29)^	Factors associated with caregiver burden among adult (19–64 years) informal caregivers – An analysis from Dutch Municipal Health Service data.	Cross-sectional descriptive study	north=1,289	Amsterdam, Netherlands (2020)	SCOPUS	CASPe 10/10
15	Mas-Casadesús O, de la Torre-Pérez L, Reig-Garcia G, Mas-Casadesús A, Berenguera A, Juvinyà-Canal D^(30)^	Building community engagement with caregivers through online interaction and a salutogenic approach in a period of isolation.	Qualitative with a socioconstructivist and phenomenological approach, and intentional sampling.	7 caregivers	Spain (2024)	SCOPUS	CASPe 9/10
16	Alonso-Rodríguez ML, Chaparro-Díaz L, Carreño-Moreno S^(31)^	Soledad, ansiedad, depresión y adopción del rol del cuidador de personas con enfermedad crónica en San Gil, Colombia.	Descriptive, analytical cross-sectional study.	115 Caregivers	Colombia (2023)	SCOPUS	CASPe 10/10
17	Haugland BSM, Hysing M, Sivertsen B^(32)^	Study progress, recreational activities, and loneliness in young adult carers: a national student survey.	National survey of higher education students in Norway, initiated by the three largest student welfare organizations [Sammen (Bergen and surrounding areas), Sit (Trondheim and surrounding areas), and SiO (Oslo and Akershus)	40,205 participants, aged 18 to 25.	Norway (2022)	SCOPUS	CASPe 10/10
18	Guets W, Perrier L^(33)^	Determinants of the need for respite according to the characteristics of informal carers of elderly people at home: results from the 2015 French national survey	Nationally representative survey, Capacités Aides et Resources des Seniors (CARE - ménage), collected in 2015, Studies, Evaluation, and Statistics (DREES).	6,201 informal caregivers (≥16 years old)	France (2021)	SCOPUS	CASPe 10/10
19	Lwin M, Sheldenkar A, Panchapakesan C^(34)^	A Digital Mobile Community App for Caregivers in Singapore: Predevelopment and Usability Study.	Pre-development survey to identify the specific needs of caregivers and gaps in currently available web-based community networks	103 caregivers	Singapore (2021)	SCOPUS	CASPe 9/10
20	van Woerden HC, Angus N, Kiparoglou V, Atherton I, Leung J^(35)^	Long-Term Conditions in Older People are Linked with Loneliness, but a Sense of Coherence Buffers the Adverse Effects on Quality of Life: A Cross-Sectional Study.	Cross-sectional survey of a random sample of 3,000 households in a defined area in northern Scotland (NHS Highland).	1,471 surveys	Scotland (2021)	SCOPUS	CASPe 10/10
21	Rippon I, Víctor CR, Martyr A, Matthews FE, Quinn C, Rusted JM, et al.^(36)^	Perspectivas diádicas sobre la soledad y el aislamiento social entre personas con demencia y cuidadores conyugales: hallazgos del programa IDEAL, Aging & Mental Health	Qualitative study, semi-structured interviews were conducted.	11 caregivers	England, Scotland, and Wales (2023)	PUBMED	CASPe 10/10
22	Ross A, Pérez A, Wehrlen L, Lee LJ, Yang L, Cox R, et al.^(37)^	Factors influencing loneliness in cancer caregivers: A longitudinal study	Prospective study of repeated measures.	129	United States (2020)	PUBMED	CASPe 10/10
23	Fee A, McIlfatrick S, Ryan A^(38)^	Examining the support needs of older male spousal caregivers of people with a long-term condition: A systematic review of the literature.	Systematic searches	Eligibility criteria were applied to the full texts of 104 articles, and 11 articles met the inclusion criteria.	United Kingdom (2020)	SCOPUS	CASPe 9/10
24	Henriques NL, Silva JB, Charepe ZB, Braga PP, Duarte ED^(39)^	Factors that promote and threaten Hope in caregivers of children with chronic conditions	Qualitative, descriptive, exploratory study	46 family caregivers	Brazil (2023)	SCOPUS	CASPe 10/10
25	Mildrum Chana S, Álvarez L, Poe A, Bibriescas N, Wang DH, DiFiglia S, et al.^(40)^	The Daily Experiences of Hispanic and Latinx Dementia Caregivers Study: Protocol for a Fully Remote Daily Diary Observational Cohort Study.	This longitudinal study on multiple time scales	500 Hispanic American caregivers	Hispanic America (2024)	PUBMED	CASPe 9/10
26	Hajek A, Kretzler B, König HH. Review^(41)^	Loneliness and Social Isolation: A Systematic Review.	Systematic review	12 studies from North America and Europe	North America and Europe (2021)	PUBMED	CASPe 9/10
27	Manskow US, Sigurdardottir S, Røe C, Andelic N, Skandsen T, Damsgård E, et al.^(42)^	Factors Affecting Caregiver Burden 1 Year After Severe Traumatic Brain Injury: A Prospective Nationwide Multicenter Study.	Prospective national multicenter study	92 caregivers	Norway (2015)	SCOPUS	CASPe 9/10
28	Manrique-Anaya Y, Barrios-Puerta Z, Chaparro-Díaz L, Carreño-Moreno SP^(43)^	Adopción del rol y soledad asociado al cuidador familiar de personas con enfermedad crónica.	Cross-sectional analytical study	390 caregivers	Colombia (2024)	SCOPUS	CASPe 10/10
29	Ris I, Schnepp W, Mahrer Imhof R^(44)^	An integrative review on family caregivers' involvement in care of home-dwelling elderly	Integrative review	26 studies	Germany (2019)	PUBMED	CASPe 9/10
30	Vasileiou K, Barnett J, Barreto M, Vines J, Atkinson M, Lawson S, et al.^(45)^	Experiences of Loneliness Associated with Being an Informal Caregiver: A Qualitative Investigation. Front Psychol	Mixed method	16 caregivers	United Kingdom (2017)	PUBMED	CASPe 10/10

The research allowed us to define the operational structure of the phenomenon of loneliness in caregivers of people with NCDs, its multidimensional origin, trajectory from the identification of vulnerability factors or antecedents, attributes, and consequences, in order to confirm its complexity, as illustrated in Chart 3.

**Chart 3:** Essential structure of the analysis of the concept of “loneliness” in caregivers of people with NCDs, 2024 (N = 30)

Background	Attributes	Consequences
**Physical:** age (young/mature adult), female gender, self-perception of poor health, time spent on caregiving >20 hours, level of patient dependency^(18,21,22,33,35-37)^.	**Perception of emotional loneliness** **Physical:** fatigue, physical discomfort, muscle pain, headache, living alone^(18,19)^, widowhood^(20)^	**Physical:** feeling of malaise, frustration, discomfort, exhaustion, fatigue, emptiness, chronic stress, pain, physical strain^(18,21,23-25,27-29,32-35,38,41,42,45)^.
**Psychological:** poor interpersonal relationships, limited coping resources^(18,21,22,33,35-37)^, caregiver stress, overload, low self-esteem, pessimistic personality^(18,25,35,37)^.	**Perception of emotional loneliness** **Psychological:** pessimistic personality^(21)^, overload, stress associated with caregiving, anxiety, and depression^(22-30)^; these feelings were part of the perception of emotional loneliness.	**Psychological:** distress, depression, anxiety, irritability, mental health problems, tension in personal relationships^(18,21,23-25,27-29,32-35,38,45)^, and anxiety^(30)^.
**Social:** living alone^(18,21,22,33,35-37)^, mandatory leave from work^(27)^, inability to participate in ordinary relationships and activities^(30)^, low family and institutional support^(3,8)^, expectations of healthcare staff and family members^(19,27,28,32,33,38-44)^.	**Perception of social loneliness** **Social:** feeling isolated, alone, unsupported, uneasy due to the loss of one’s social network^(3,27)^, poor relationships with family and friends^(8,31)^, feelings of exclusion, isolation, social overload^(32,33)^	**Social:** interferes with social role performance, can trigger loss of relationships with friends, family, and social life, negative effects with feelings of frustration^(29,42)^, and loneliness that prevents taking on new roles over time^(31)^.
**Spiritual:** fatigue, hopelessness, low self-efficacy and self-efficacy, uncertainty, emotions that increase feelings of loneliness^(20,28,29,33,34,38,39)^.	**Perception of social loneliness** **Spiritual:** difficulty asking for help and finding like-minded people^(34)^, low sense of coherence, high levels of social loneliness^(35)^, poor social skills^(20)^.	**Spiritual:** uncertainty, unclear life plans, and hopelessness^(18,21,23-29,32-35,38,39)^.

1. Selection of the concept of “loneliness” in caregivers of people with NCDs

Analyzing the concept of loneliness is significant in science and nursing practice. It is justified by its increase during the Covid-19 pandemic, defined as a global public health problem, as it is a risk factor for illness and for compromising overall health^(3)^.

2. Purpose of analyzing the concept of “loneliness” in caregivers of people with NCDs.

Analyzing the concept of loneliness in caregivers of people with NCDS represented a broad and systematic exploration of the concept, based on an understanding of the phenomenon to guide the practice of care among informal caregivers.

3. Identification of the uses of the concept of “loneliness” in caregivers of people with NCDs.

In line with the literature, the concept is a phenomenon experienced by all individuals throughout their lives, likely to be perceived^(8)^ by caregivers as emotional and social loneliness.

4. Defining characteristics or attributes of the concept of “loneliness” in caregivers of people with NCDs.

The findings allowed us to describe the properties of perceived loneliness in terms of physical, emotional, social, and spiritual suffering. In the physical and emotional dimensions, these included manifestations of fatigue, physical discomfort, muscle pain, headache, living alone^(18,19)^, widowhood^(20)^, pessimistic personality^(21)^, overload, stress associated with caregiving, anxiety, and depression^(22-30)^; these feelings were part of the perception of emotional loneliness.

In relation to social and spiritual suffering, they reported feeling isolated, alone, unsupported, uneasy due to the loss of their social network^(3,27)^, low levels of relationship with family and friends^(8,31)^, feelings of exclusion, isolation, social overload^(32,33)^, difficulty asking for help and finding like-minded people^(34)^, low sense of coherence, high levels of social loneliness^(35)^, low social skills^(20)^, context linked to the perception of social loneliness, as shown in Chart 3.

5. Identify the background of the concept of “loneliness” in caregivers of people with NCDs

Loneliness among caregivers is generated by multiple causes; the vulnerability described in the literature highlighted physical, psychological, social, and spiritual factors that predict the perception of loneliness related to caregiving, as illustrated in Chart 3.

Physical: age (young/mature adult), female gender, self-perception of poor health, time spent on caregiving >20 hours, level of patient dependency^(18,21,22,33,35-37)^.

Psychological: poor quality interpersonal relationships, poor coping resources^(18,21,22,33,35-37)^, caregiver stress, overload, low self-esteem, pessimistic personality^(18,25,35,37)^.

Social: living alone^(18,21,22,33,35-37)^, retirement/mandatory leave from work^(27)^, inability to participate in ordinary relationships and activities^(30)^, low family and institutional support^(3,8)^, expectations of health personnel and family members^(19,27,28,32,33,38-44)^.

Spiritual: fatigue, hopelessness, low self-efficacy and self-efficacy, uncertainty, emotions that increase feelings of loneliness^(20,28,29,33,34,38,39)^.

6. Consequences related to the concept of “loneliness” in caregivers of people with NCDs.

Loneliness arises as a consequence of providing care at home and is also considered a predictor of suffering, with consequences for well-being.

Physical: feeling of malaise, frustration, discomfort, exhaustion, fatigue, emptiness, chronic stress, pain, physical burden^(18,21,23-25,27-29,32-35,38,41,42,45)^.

Psychological: distress, depression, anxiety, irritability, mental health problems, tension in personal relationships^(18,21,23-25,27-29,32-35,38,45)^, and anxiety^(30)^.

Social: interferes with social role performance, potentially triggering loss of relationships with friends, family, and social life; negative effects with feelings of frustration^(29,42)^ and loneliness that prevent taking on new roles over time^(31)^.

Spiritual: uncertainty, confused life plans, and hopelessness^(18,21,23-29,32-35,38,39)^.

Chart 3 summarizes the structure of the concept of loneliness in caregivers of people with NCDs, presenting antecedents, attributes, and consequences.

**Figure 2:**
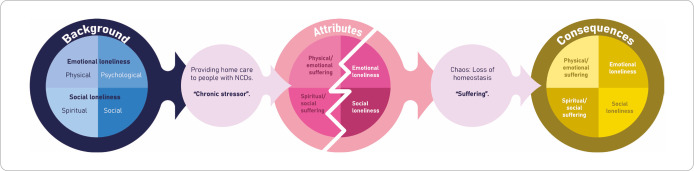
Contextual framework for analyzing the concept of “loneliness” in caregivers of people with NCDs, 2024

Based on the results obtained in the synthesis of the different investigations, it was possible to reflect on the trajectory of caring for people with NCDs, from the background, the dynamic nature of care over time, and the effects and impacts on all areas of the lives of caregivers of people with NCDs, a process that brought greater clarity to the contextual framework of this phenomenon, as shown in Figure 2.

7. Define the empirical references of the concept of “loneliness” in caregivers of people with NCDs.

This step is essential to present how to measure or quantify concepts in reality. The most common symptom manifested among caregivers was overload, but loss of personal space and feelings related to anxiety and loneliness also stood out.

8. Model case of the concept of “loneliness” in caregivers of people with NCDs.

The defining attributes of loneliness in caregivers are evident: Alicia is 55 years old and has been caring for her husband, Jaime, who was diagnosed with Alzheimer’s at age 52, for eleven years. She describes herself as a hard-working person with a vocation for helping others. She lives with Jaime and two sisters-in-law, none of whom are employed. Since her husband fell ill, Alicia has had to give up her business to devote herself fully to caring for him and supporting her family. This situation has caused serious financial difficulties. Jaime’s health has progressively deteriorated, and he was recently diagnosed with cancer. In addition, Alicia faces her own health problems, which aggravates her situation.

Initially, Alicia noticed subtle changes in her husband’s behavior, although she was unaware of the disease. She noticed unusual situations, such as reports of experiences in places he had never been, the loss of objects that he later attributed to her or her sisters. To better understand what was happening, she began to read books on the subject, which allowed her to understand the disease. The initial phase was particularly difficult for her, causing her to cry on numerous occasions. As Alzheimer’s progressed, Jaime developed a tumor in his nose, for which he underwent four surgeries until it was confirmed to be malignant. In October last year, Alicia discovered a lump in her husband’s armpit, and after a biopsy, another cancer diagnosis was confirmed.

Jaime is currently facing two complex illnesses, while Alicia is experiencing profound emotional exhaustion. According to the doctor, her husband is in the third stage of Alzheimer’s and is still able to move around on his own. However, she feels exhausted, isolated, and full of uncertainty about the future. She often retreats to cry so that others won’t see her and expresses a feeling of emptiness.

Financial resources are insufficient, as Alicia does not have her own pension and her husband’s pension does not cover all expenses. Although the family remains united, living together has become difficult. Her sisters-in-law, for example, show discomfort with everyday situations involving Jaime’s decline, such as when he spills food or has difficulty controlling his secretions.

As for her own health, Alicia has had spinal surgery and has a plate in her cervical vertebrae. She also suffers from fibromyalgia and is awaiting another medical procedure. She often wakes up wanting to cry because of the constant pain, which prevents her from sleeping well. Despite this, she continues to share a bed with Jaime, as he experiences fears, hallucinations, and sees non-existent insects, which inspires compassion in her. However, her husband’s sudden movements during the night force her to seek rest on the sofa until dawn, getting only a few hours of sleep.

Emotionally, Alicia experiences constant ups and downs. The previous week, for example, she felt particularly depressed, avoiding contact with her sisters-in-law and preferring to be alone. When the house is quiet, she cries silently to relieve herself. She cannot take sleeping pills because she needs to stay alert for her husband. She feels alone in her pain and unsupported, which has led her to isolation. She misses the man her husband used to be, an attentive and loving companion, and it is deeply painful to see him fade away little by little.

9. Related case of the concept of “loneliness” in caregivers of people with NCDS.

Some attributes of the concept of loneliness are presented: Carmenza, a 60-year-old woman, comments to her daughter: “I didn’t decide to be Grandma’s caregiver, it just fell to me.” She began to notice deterioration and forgetfulness in her mother; she was the one who was closest to her. Although Miguel, another son, lived a few blocks away, he was a man. Carmenza asks herself: “Why wasn’t he the caregiver?” Because he is a man. Because he had his home and his things...” “That’s a tough question,” she says. She thinks that women are more available to be caregivers. No one told her to be a caregiver. Life led her to it, and she didn’t give up. She had no choice. They [her brothers] never thought about taking responsibility for their parents.

Carmenza doesn’t believe it’s just because she’s a woman, because her sister is a woman and it wasn’t her responsibility. She thinks that people become caregivers because they have a different sensitivity than others, and they take advantage of that. The caregiver decides to be there, and then they can’t leave. She thinks, “I’ll do this for today,” but when she realizes it, she is alone with all the responsibility, alone, isolated; she had to stop working and shut herself away with her parents, and she couldn’t get out of that maze.

She says: there are roles she is supposed to fulfill. Her family is more aware, they are self-sufficient, and so she can leave them a little to one side and take on the front line of caring for her mother. She has had problems because, before this situation, she had agreed on certain roles with her husband, and now they need to be changed; when she is on good terms with her husband, she may be better off there with her mother.

## DISCUSSION

This conceptual analysis highlights the complexity of the phenomenon described; loneliness in caregivers is the outcome of several intertwined circumstances that people may experience at some point in certain situations and special conditions^(1,22,23)^. Currently, loneliness is considered a problem and a challenge for public health due to its relationship with low well-being and compromising quality of life among caregivers, who perceive it as an unpleasant feeling that favors negative health states, conditions that increase blood pressure, markers of inflammation, cause cognitive deterioration, organic dysfunctions, psychophysiological disorders, and chronic diseases, which together can increase mortality^(13)^.

It is crucial to differentiate between loneliness as a subjective experience of emotional isolation and social isolation as an objective situation of lack of social contact. While isolation is an objective condition in which a person is physically alone but may feel connected to others, loneliness is subjective and can be experienced even in the presence of other people. The latter state is often painful and distressing^(48)^.

The functional decline and dependence of people with chronic noncommunicable diseases (CNCDs), coupled with the isolation imposed during the pandemic, have accentuated the perception of loneliness among caregivers. Despite sharing their homes with other family members, many caregivers feel that the responsibility for care falls solely on them. This feeling has intensified due to the limited presence of health professionals in homes during the health crisis^(24,30)^.

Loneliness among caregivers is defined as the feeling of being alone, without anyone to talk to, misunderstood, in need of company, of being considered^(25,31,32,34)^; perception of distress due to lack of social connection^(26)^; feeling of social isolation resulting from the demands of care^(18,27,33,35,36)^. Those who care without family and institutional support experience the complex emotional and social situation of loneliness in response to the stress involved in caregiving, as well as the financial and emotional impact^(19,28)^.

Loneliness is the psychological condition resulting from the discrepancy between existing social relationships and those desired by the individual^(20)^. It is a feeling perceived as real pain, defined in physical terms as emotional loneliness and, in terms of isolation, as social loneliness, which can affect physical and psychological health and family functioning^(21,29)^. In particular, loneliness has a negative impact on the emotional well-being of male caregivers, accompanied especially by a feeling of isolation^(38)^.

The Royal Spanish Academy defines loneliness as the voluntary or involuntary absence of company and as a feeling of melancholy derived from the loss or absence of someone or something^(46)^. On the other hand, Peplau and Perlman explain that loneliness arises from an imbalance between an individual’s actual social relationships and their expectations of them^(47)^.

It is important to differentiate between social isolation and loneliness. While isolation is an objective condition in which a person is physically alone but may feel connected to others, loneliness is subjective and can be experienced even in the presence of other people. The latter state is often painful and distressing^(48)^.

Among the concepts most commonly used to describe caregiver loneliness, the following were identified: feelings of emotional distress, pain, emptiness, suffering, anguish, hurt, helplessness, sadness, desolation, discomfort, hopelessness, defenselessness; psychological conditions resulting from the discrepancy between existing social relationships and those desired by the individual^(20)^. Two types of loneliness were identified: emotional loneliness, which causes feelings of emptiness, abandonment, and desolation due to the absence of meaningful interpersonal relationships, and social loneliness, in which the lack of friends, siblings, and neighbors is recognized^(49)^.

Based on this review of the use of the concept of “loneliness” in caregivers, it can be said that it is a negative and undesirable feeling, perceived as painful and real, defined in physical terms as emotional loneliness and, in terms of isolation, as social loneliness, with important effects on physical and psychological health and family functioning^(21,29)^.

In the literature review, the attributes that best explain the concept of loneliness in caregivers can be summarized in two dimensions: emotional loneliness and social loneliness.

Emotional loneliness is defined as a feeling of tiredness, physical discomfort, muscle pain, headache, low back pain, and being alone^(18)^, sometimes resulting from a personal decision^(19)^ or life transitions, such as widowhood, living alone, or living in a remote rural area^(20)^. It is associated with pessimistic personality traits in relation to others and oneself^(21)^, high levels of overload, stress associated with caregiving, anxiety, depressive symptoms, manifestations of lack of time for self-care, and basic role adoption^(22-29,40)^.

Social isolation is perceived as feeling isolated, alone, unsupported, uneasy about giving up important aspects of one’s life^(30)^, poor quality relationships with other family members and friends^(31)^, having few close friends or people to talk to. Caregivers reported deep feelings of exclusion and isolation, especially young people with more extensive caregiving responsibilities^(32)^, who live with the person being cared for and devote more than 30 hours per month to caregiving^(33)^.

Caregivers face great difficulties in finding people with similar experiences^(34)^, which contributes to a low sense of coherence and an increased perception of social loneliness^(35)^. They may also have limited social skills^(21)^, which further hinders the possibility of establishing supportive bonds.

From a temporal perspective, loneliness can be classified as follows^(50)^:

- Chronic loneliness: when a person, regardless of their surroundings, feels persistent isolation for years.
- Situational loneliness: occurs at specific times, such as mourning after the loss of a loved one.
- Transient loneliness: results from specific events and is temporary, such as interpersonal conflicts.

According to the literature, the predictive factors for the perception of loneliness were: age (young/mature adult), female gender, low levels of education, poor social relationships, poor self-assessment of health, decreased functionality of the person being cared for, multiple care actions performed, perception of help received, living alone, more than 20 hours of unpaid care^(18,21,22,33,38,41)^.

The high level of demand in the caregiver role, combined with social isolation and lack of support, can lead to changes in the relationship with the person being cared for^(42-45)^. In addition, reduced participation in support networks contributes to stress, overload, and decline in emotional well-being^(19,26,36,37)^. In many cases, this situation leads to abandonment of paid employment and can cause feelings of confinement and hopelessness^(27)^, scarce coping resources^(22-25,31)^, which translates into the inability to participate in ordinary relationships and activities^(30)^.

The lack of family and institutional support, as well as the expectations of healthcare professionals and family members, are a source of stress and changes in social roles. In this context, feelings of hopelessness increase, along with doubts about self-efficacy and uncertainty about the future; thus intensifying feelings of loneliness, a significant need for respite care^(20,28,29,33,34,38,39)^, with an inability to “solve” the daily problems faced by the sick person, helplessness, conflictual interpersonal relationships, and a sense of exclusive responsibility that increase frustration and suffering^(51)^.

Negative effects, feelings of frustration^(30)^, and the inability to take on new roles over time^(31)^ arise as consequences related to “loneliness”.

Harmful effects on the caregiver’s health and well-being were also evident, with manifestations of: grief, helplessness, sadness, desolation, discomfort, malaise, frustration, exhaustion, fatigue, emptiness, chronic stress, pain, physical burden, distress, depression, anxiety, irritability, mental health problems, uncertainty due to lack of clarity in life plans, hopelessness, tension in personal relationships^(18,21,23-25,27-29,32-35,38)^, physical and emotional suffering, increased morbidity due to hypertension and diabetes mellitus, increased inflammatory markers, and mortality^(26)^.

The expectations of health professionals and family members^(19)^ can add an additional burden, causing some caregivers to feel unable to continue their task^(23)^. In addition, the impact of the burden of care varies according to gender. In the case of men, taking on this role may conflict with their masculine identity, due to the traditional perception of care as a female task^(38)^.

Caregivers often experience symptoms such as stress, loss of personal space, and anxiety, as well as a deep sense of loneliness^(30)^ and feeling alone. One of the most widely used scales worldwide to measure loneliness is the University of California, Los Angeles (UCLA) Loneliness Scale^(19,20,22-25,26,31,32)^, employed for its unidimensional structure, in the version validated in Spanish, and tested in the adult population^(51)^; low scores indicate greater loneliness. The scale measures the perception of loneliness at low, moderate, and severe levels^(51)^.

Another widely used tool is the De Jong Gierveld Loneliness Scale (De Jong Gierveld & Van Tilburg, 1999)^(21,29,35,36)^, applied in several international studies.

The empirical composite health status index captures caregiver vulnerability (HSCIi)^(33)^ and has been used to assess the likelihood of experiencing loneliness. It includes questions about demographics, health problems of the care recipient, mental and physical health problems of the caregiver, use of digital media, search for information and support, to identify problems related to care, support provided, and what caregivers would want from a mobile care application^(34)^; a high coherence index is related to a lower probability of experiencing loneliness.

### Study limitations

The main limitation of this concept analysis is related to the search period, considering that new publications appear daily in different databases, the geographical location of the studies, which makes them difficult to access, and the restriction on accessing full-text articles in databases. The authors controlled for these limitations by searching over a 10-year period; databases with access to full articles, different languages, and geographic regions were used. Possible risks and biases were controlled by eliminating gray literature and restricted-access articles; in addition, the articles were evaluated independently by the authors (CPCM, MVOL, VMS, RGB, JFVC, and CARP). Although there is consensus in the literature on the perception of loneliness among caregivers, it has been studied from various disciplines that incorporate an extensive collection of scientific products. By integrating the antecedents, attributes, and consequences of the concept under study, a clearer expression is achieved.

The findings of this study allow us to understand loneliness among caregivers as a complex and multidimensional phenomenon, resulting from the interaction between caregiving demands, personal conditions, family dynamics, and insufficient support networks^(18-36)^. These aspects are consistent with previous investigations that document caregiver vulnerability^(18-22,27-29,32,33,35-44)^ and emphasize the need for comprehensive interventions. The literature review also showed that loneliness has a cross-cutting impact on all dimensions of well-being, which affects the caregiver’s ability to sustain prolonged care processes.

With regard to the regionality of scientific production, a concentration was identified in Europe, predominantly in the United Kingdom and, to a lesser extent, in Norway, Germany, Spain, France, the Netherlands, and Turkey, confirming the consolidated research trajectory in this region. In the Americas, contributions came from Colombia, the United States, Mexico, Cuba, Chile, and Brazil, highlighting the participation of both North America and Latin America and positioning Colombia as a regional benchmark. In Asia, studies were located in Iran, China, and Singapore, with a more dispersed but still relevant distribution. In contrast, the absence of publications in Africa and Oceania reveals gaps in the global approach to the phenomenon and points to the need to expand research in these regions in order to promote a broader and more diverse understanding.

### Contributions to nursing, healthcare, or public policy

This research is of fundamental importance to the discipline of nursing; it allows for the understanding of a phenomenon of interest to the care of people in chronic situations and their caregivers. Its focus enables an approach to the concept of loneliness among caregivers. The study’s findings reveal multiple characteristics that guide how the phenomenon under study can be modified through the formulation and implementation of public policies and scientifically tested interventions to prevent loneliness and/or minimize it when already present, thus contributing to achieving better physical, psychological, social, and spiritual well-being.

## CONCLUSIONS

This study revealed the complexity of the concept of “loneliness” among caregivers of people with NCDs, a phenomenon that is universally experienced and perceived in a personal and subjective manner. The focus on the topic based on conceptual analysis allowed us to identify antecedents of vulnerability and the complexity of caregiving conditions, characterize attributes, and indicate consequences for caregivers. The literature review presented evidence that guides how the phenomenon studied can be avoided and/or modified based on clinical reasoning in light of the real needs of caregivers of people with NCDs, allowing for the proposal of policies and implementation of therapeutic plans aimed at preventing and reducing loneliness among caregivers of people with NCDs.

## Data Availability

The research data are available within the article.
