# Supplementary material for: Loneliness in caregivers of people with chronic illnesses: conceptual analysis according to Walker and Avant
Source: Rev Bras Enferm. 2026 Feb 23;79:e20240651. doi: 10.1590/0034-7167-2024-0651 (PMC12933932; doi:10.1590/0034-7167-2024-0651)
Supplement: Translation

# Translation: Soledad en los cuidadores de personas con enfermedades crónicas: Análisis conceptual según Walker y Avant

**DOI:** 10.1590/0034-7167-2024-0651es

**Authors:** Claudia Patricia Cantillo Medina, Marcos Venícios de Oliveira Lopes, Viviane Martins Silva, Reinaldo Gutierrez Barreiro, Janaina Fonsecca Victor Coutinho, Claudia Andrea Ramírez-Perdomo

**Affiliations:** 1 Universidad Surcolombiana. Neiva, Huila, Colombia.; 2 Universidad Federal de Ceará. Fortaleza, Ceará, Brasil.

**Keywords:** Cuidadores, Enfermedad Crónica, Formación de Conceptos, Investigación en Enfermería, Soledad

## Abstract

**Objetivos:**

analizar el concepto de soledad en los cuidadores de personas con enfermedad crónica no transmisible.

**Métodos:**

análisis de concepto planteado por Walker y Avant. Se incorporó un estudio cuantitativo tipo revisión de alcance para identificar la bibliografía científica disponible basada en el marco teórico del Instituto Joanna Briggs, incluyó 30 artículos de las bases de datos Scopus y PubMed, publicados entre 2015 al 2024 en español, inglés y portugués con puntaje mayor o igual a 8/10 según el Critical Appraisal Skills Programme Español.

**Resultados:**

los antecedentes, atributos, y consecuencias identificadas del análisis conceptual de “soledad” en el cuidador, permitieron describir los efectos del cuidado en las dimensiones del bienestar físico, emocional, social, y espiritual.

**Conclusiones:**

se reveló la complejidad y trayectoria multidimensional del fenómeno de soledad, la cual permite proponer políticas dirigidas a su prevención y aportar al bienestar de la población.

## INTRODUCCIÓN

La soledad es un fenómeno universal, experimentado por todos los individuos durante su vida, su aumentó en la prevalencia durante el brote por Covid-19, la definió como un problema de salud pública mundial^(1)^. Junto a este fenómeno se han aumentado a nivel mundial las enfermedades crónicas no transmisibles (ECNT), las cuales ocasionan disminución en la funcionalidad y dependencia de cuidado; condiciones que afectan específicamente al individuo, familias y cuidadores. Por esta razón son consideradas importantes causas de sufrimiento, y discapacidad con una significativa carga económica y social^(2)^.

En Europa, diferentes países del norte y el oeste reportaron una prevalencia del sentimiento de soledad entre el 10% y el 20% en las personas cuidadoras, incrementándose hasta aproximadamente el 25% durante los cinco primeros meses del brote por Covid-19. En los países de Europa del Este, los valores de prevalencia observados fueron superiores, oscilando entre el 30% y el 55%^(1)^. En Reino Unido 21%, en 28 países de África, Asia y las Américas, países de ingresos bajos y medianos, se observaron tendencias crecientes significativas^(1)^.

Quienes cuidan generalmente son integrantes de la familia, amistades, o vecinos que proporcionan asistencia sin remuneración a otros con limitación física, psicológica o cognitiva, ellos generalmente resultan inesperadamente comprometidos en su rol. Deben asistir a la persona cuidada en su vida cotidiana, suministrar medicación, utilizar equipos, organizar asistencia a controles, decidir y resolver situaciones de bienestar y salud, muchas veces sin la preparación adecuada para la complejidad de la salud y vida de su ser querido en el contexto domiciliario^(3)^.

Debido a la demanda de atención, y tiempo invertido en el desempeño, cuidar se considera un estresor crónico a nivel psicológico, y se asocia con la presencia de sintomatología ansiosa, depresiva, sobrecarga y soledad^(4-6)^. En la literatura se evidencia asociación entre el estrés objetivo y subjetivo con el cuidado diario, que contribuyen directamente a que emerjan sentimientos de soledad, afines a mayor presentación de sintomatología depresiva^(7)^. Los cuidadores a menudo se debaten entre perjuicios, privaciones y distanciamiento de sus círculos sociales, reducción del espacio personal y relaciones interpersonales^(8)^, circunstancias que intensifican los sentimientos de soledad^(9)^.

En los cuidadores con disponibilidad de apoyo social existe mejor cumplimiento del rol y actividades de autocuidado, mayor empoderamiento y habilidad para utilizar recursos disponibles, además disminuyen los sentimientos de soledad y problemas psicológicos^(10)^. La percepción de soledad se define como una experiencia dolorosa que ocurre cuando las redes sociales son deficientes, las personas sufren soledad al percibir déficit en sus relaciones; por tener un menor número al deseado o cuando estas no aportan la intimidad que necesitan^(11)^.

La soledad percibida por los cuidadores, se describe como la diferencia entre las expectativas de las relaciones interpersonales para con los demás, en referencia a la calidad o veracidad de las conexiones sentidas frente a su realidad^(11)^. Su presentación crónica puede ser causa de mortalidad prematura, y enfermedades crónicas^(12)^. Desde la perspectiva biopsicosocial, la disponibilidad de relaciones sociales es una herramienta para la promoción de la salud y el bienestar^(13)^. Comprender el concepto de soledad en los cuidadores de personas con ECNT es una importante aproximación a la brecha conceptual frente a esta realidad, y puede servir de base para plantear políticas e implementar estrategias de intervención orientadas hacia los cuidadores informales en la prevención de la soledad.

## OBJETIVOS

Analizar el concepto de “soledad” en los cuidadores de personas con ECNT.

## MÉTODOS

El estudio siguió un enfoque teórico mediante el método de análisis de concepto de Walker & Avant, conocido por su rigor y precisión, consta de ocho pasos^(14)^:

1. Seleccionar el concepto: se determina para clarificar la definición del fenómeno de estudio con el fin de evitar el uso inexacto en la teoría y la investigación de enfermería^(14)^.
2. Determinar el propósito del análisis: representa la exploración del concepto de manera amplia y sistemática, fundamentada en la comprensión del fenómeno como base para generar políticas, estrategias e intervenciones que guíen la práctica del cuidado^(14)^.
3. Identificar los usos del concepto: establece las formas en que se emplea el concepto, y revela los atributos descriptivos del fenómeno^(14)^.
4. Determinar las características o atributos definitorios del concepto: cada concepto contiene más de un atributo definitorio, se repiten una y otra vez, son clave en su diferenciación con otros; es necesario determinar qué atributo es más apropiado para describirlo^(14)^.
5. Identificar los antecedentes del concepto: comprenden las posibles circunstancias, contextos o eventos que ocurren antes del concepto^(14)^.
6. Consecuencias del concepto: son sucesos o resultados que pueden ocurrir después del concepto o como producto del concepto^(14)^.
7. Definir los referentes empíricos: muestran los métodos para medir conceptos y hasta qué punto la definición puede ser útil para medir y validar el concepto, asimismo producir herramientas de investigación^(14)^.
8. Identificar un caso modelo e identificar casos relacionados: el modelo o caso es un ejemplo puro del concepto en estudio y debe tener todos los atributos definitorios^(14)^, y el relacionado muestra un ejemplo del concepto en estudio que representa algunos de los atributos definitorios^(14)^.

Para obtener datos e identificar los elementos del análisis de conceptos, se realizó un estudio cuantitativo tipo una revisión de alcance basada en el marco teórico del Instituto Joanna Briggs (JBI)^(15)^, se adoptó la recomendación del protocolo establecido por la Extensión de Elementos de Informe Preferidos para Revisiones Sistemáticas y Metaanálisis para Revisiones de Alcance (PRISMA-ScR)^(15)^. En el proceso de revisión se siguieron los lineamientos propuestos por Arksey, O’Malley^(16)^ utilizados para identificar los conceptos clave que sustentan un campo de investigación, así como para aclarar definiciones de trabajo y/o los límites conceptuales de un tema. Esto implicó identificar las fuentes clave de evidencia, sintetizar y mapear las características principales de los estudios incluidos, como los diseños de investigación utilizados, las poblaciones objetivo, las intervenciones o exposiciones evaluadas, y los resultados reportados^(16)^.

La pregunta de investigación que guio la revisión utilizó la estrategia PCC, en este sentido se estructuro de la siguiente manera: (P): Cuidadores familiares de personas con enfermedad crónica no transmisible, (C): Concepto de Soledad, (C): Mundial.

Así la pregunta orientadora fue: ¿Cuáles son los usos, atributos, antecedentes, consecuencias y referentes empíricos del concepto de soledad en los cuidadores de personas con ECNT?.

El proceso de revisión y verificación incluyó cinco pasos:

Paso 1: Búsqueda en bases de datos.

El protocolo de revisión desarrollado por los investigadores en las bases de datos de Scopus y PubMed, de julio a septiembre del 2024, se utilizaron los operadores boléanos “AND” y “OR”, que relacionaron los siguientes términos de búsqueda DeCS-MeSH combinando las palabras clave: Cuidadores; Enfermedad Crónica; Formación de concepto; Investigación en enfermería; Soledad, la ecuación fue ajustada en cada una de las bases de datos (Cuadro 1).

**Cuadro 1:** Términos de búsqueda con descriptores DeCS y MeSH

Bases de datos	Ecuación de búsquedas en inglés	Resultados
Scopus	“Chronic Disease” OR “Noncommunicable Diseases” AND “Caregivers” OR “Informal Caregivers” OR “familiar Caregivers” AND “Loneliness” OR “Social Alienation”	20
PubMed	((((“Chronic Disease”[Mesh]) OR “Noncommunicable Diseases”[Mesh]) AND “Caregivers”[Mesh]) AND “Loneliness”[Mesh]) OR “Social Alienation”[Mesh]	10

Paso 2: Revisión de criterios de inclusión.

Los artículos elegidos fueron los escritos en español, inglés y portugués, publicados entre 2015 al 2024, estudios cuantitativos y cualitativos publicados en revistas indexadas, incluyeron investigaciones experimentales, descriptivas, ensayos clínicos, estudios piloto, y cualitativos. Se recopilaron los artículos en el programa Microsoft Excel® en una matriz en la que se definieron categorías de información, se criticó la calidad metodológica del artículo por medio del Critical Appraisal Skills Programme Español (CASPe)^(17)^, cuyo objetivo es proporcionar las habilidades necesarias para la “lectura crítica de la evidencia clínica”, se seleccionaron los artículos que obtuvieron un puntaje mayor o igual a 8/10. Se excluyó literatura gris, cartas, comentarios, opiniones de expertos y editoriales.

Paso 3: Análisis de datos.

Cada estudio se analizó individualmente y los datos relevantes se incorporaron en un cuadro descriptivo con información sobre los autores, año de publicación, ubicación, definición del concepto de soledad en cuidadores de personas con ECNT, uso, características o atributos definitorios, antecedentes, consecuencias, referentes empíricos, resultados, y conclusiones principales.

Paso 4: Verificación a texto completo.

Se realizó lectura a texto completo para verificar el aporte de cada estudio seleccionado. Se extrajeron los datos relevantes, incluyendo diseño de estudio, características de los participantes, factores identificados, resultados principales y conclusiones. Luego se resumieron de manera sistemática.

Paso 5: Resumen y presentación de los resultados

Para una mejor comprensión del fenómeno se identificaron los temas y patrones extraídos de los artículos que prevalecieron en los estudios, así como la identificación de patrones, tendencias y brechas en la literatura. Se asignaron categorías analíticas congruentes con el área de interés, y fueron agrupados temáticamente^(16)^. Este estudio no requirió aval del Comité de Ética y Bioética porque no involucró seres humanos, se respetaron los principios de derechos de autor en la utilización de referencias del material científico analizado.

## RESULTADOS

Se determinaron 650 estudios potencialmente elegibles. Fueron eliminados por criterios de selección predeterminados 370 documentos, 205 por no estar disponibles de forma gratuita y en texto completo, 6 artículos duplicados, 39 después de la revisión de títulos y resúmenes. Las referencias de los artículos y aquellos considerados relevantes en el tema de la soledad de los cuidadores fueron revisados manualmente. Se seleccionaron para lectura completa y análisis 30 artículos, de acuerdo con la Figura 1.

**Figura 1:** Diagrama de flujo PRISMA-ScR de estrategia de búsqueda y selección de artículos, en bases de datos, 2024

Se consolidaron los resultados de la revisión de alcance mediante la descripción de las características generales de los estudios seleccionados (Cuadro 2). La muestra estuvo compuesta por 30 estudios de la literatura internacional y nacional publicados en revistas indexadas entre los años 2015 y 2024 (Cuadro 2). Los artículos incluidos fueron publicados en: Reino Unido (7), Colombia (4), Estados Unidos (3), Noruega, México, Cuba (2), Alemania, España, Francia, Países Bajos, Turquía, Irán, China, Singapur, Chile, y Brasil (1).

**Cuadro 2:** Resumen de los artículos analizados en la revisión de alcance sobre análisis de concepto soledad en los cuidadores de personas con ECNT, (N=30), 2024

No	Autor	Título o referencia	Diseño /participantes	Muestra / meta	País / Año	Base de datos	CASPe
1	SerçekuÅ P. Becoming a family caregiver of a patient living with cancer^(3)^	Becoming a family caregiver of a patient living with cancer.	Autoetnografía	1	Turquía (2020)	PUBMED	CASPe 8/10
2	Victor CR, Rippon I, Quinn C, Nelis SM, Martyr A, Hart N, et al.^(8)^	The prevalence and predictors of loneliness in caregivers of people with dementia: findings from the IDEAL programme	Estudio de cohorte Improving the experience of Dementia and Enhancing Active Life (IDEAL)	1283 cuidadores familiares de personas con demencia leve a moderada que vivían en Gran Bretaña.	Gran Bretaña (2021)	PUBMED	CASPe 10/10
3	Blanco-Martínez L, Simón-Álvarez A, Sánchez-Fernández A^(18)^	Caracterización biopsicosocial de los cuidadores informales de pacientes con enfermedad crónica o terminal	Estudio descriptivo transversal	87 cuidadores de personas con enfermedad crónica	Cuba (2016)	SCOPUS	CASPe 9/10
4	Hejazi S, Manzour R, Shahsavari A, Ghasemi S, Roshan-Nejad M^(19)^	Experiences of family caregivers of individuals undergoing hemodialysis in Iran about caring during the COVID-19 pandemic: a qualitative study	Cualitativo, mediante un análisis de contenido cualitativo inductivo.	17 cuidadores familiares de personas sometidas a hemodiálisis en Bojnurd, Irán	Irán (2024)	SCOPUS	CASPe 9/10
5	Hui Y, Wang Y, Dongxia Xiao L, Liu L^(20)^	Prevalencia y factores asociados con síntomas psicológicos negativos entre viudas mayores que viven solas en una muestra remota de China: un estudio transversal.	Estudio transversal	271 Viudas	China (2023)	SCOPUS	CASPe 9/10
6	Holton E, Bernadette Boyle N, Simons R, Warters A, O’Philbin L, Lawlor B, et al.^(21)^	Freedom and loneliness: dementia caregiver experiences of the nursing home transition	Estudio cualitativo, se realizaron entrevistas semiestructuradas	11 personas cuidadores	República de Irlanda (2023)	PUBMED	CASPe 8/10
7	Carrillo Cervantes AL, Medina Fernández IA, Medina Fernández JA, Chaparro Díaz L, Carreño Moreno SP, Cortez González LC^(22)^	Soledad, ansiedad, depresión y adopción del rol de cuidador de adultos mayores con enfermedad crónica durante la covid-19.	Diseño correlacional predictivo.	157 cuidadores	México (2022)	SCOPUS	CASPe 9/10
8	Mares-Rico KF, Cardona-Ramírez VL, Franco-Álvarez DM, Medina-Fernández IA, Medina-Fernández JA, Carrillo-Cervantes AL^(23)^	Ansiedad, depresión y soledad en cuidadores de personas con enfermedad crónica.	Diseño descriptivo correlacional.	178 cuidadores familiares	México (2022)	SCOPUS	CASPe 9/10
9	Miller K; Van Houtven C, Smith V, Hoff Lindquist J, Gray K, Richardson C, et al.^(24)^	Los cuidadores familiares de veteranos experimentan niveles clínicamente significativos de angustia antes y durante la pandemia: Implicaciones para los servicios de apoyo a los cuidadores	Diseño previo y posterior y datos longitudinales	903 cuidadores	EEUU (2022)	PUBMED	CASPe 10/10
10	Carreño-Moreno S, Pacheco-López M, Arias-Rojas M^(25)^	Role adoption, anxiety, depression and loneliness in family caregivers of patients with chronic diseases	Estudio descriptivo, exploratorio y transversal	960 cuidadores familiares	Colombia (2024)	SCOPUS	CASPe 10/10
11	Valenzuela M, Slachevsky A, Thumala D, Pinto A, Olavarría L, Lema J, et al.^(26)^	Soledad en cuidadores informales de personas con demencia durante la pandemia de COVID-19	Estudio cuantitativo de carácter transversal	195 cuidadores	Chile (2023)	SCOPUS	CASPe 9/10
12	Saadi JP, Carr E, Fleischmann M, Murray E, Head J, Steptoe A, et al.^(27)^	The role of loneliness in the development of depressive symptoms among partnered dementia caregivers: Evidence from the English Longitudinal Study of Aging	Estudio Longitudinal Inglés sobre el Envejecimiento (ELSA).	10,813 personas que habían brindado cuidados a su pareja entre la oleada	Inglaterra (2021)	SCOPUS	CASPe 9/10
13	Parada-Rico DA, Carreño-Moreno S, Chaparro-Díaz OL^(28)^	Soledad, ansiedad y depresión en la adopción del rol de cuidador familiar del paciente crónico	Investigación cuantitativa analítica transversal	120 Cuidadores	Colombia (2023)	SCOPUS	CASPe 9/10
14	Koopman E, Heemskerk M, van derBeek AJ, Coenen P^(29)^	Factors associated with caregiver burden among adult (19–64 years) informal caregivers – An analysis from Dutch Municipal Health Service data.	Estudio descriptivo transversal	norte=1.289	Ámsterdam, Países Bajos (2020)	SCOPUS	CASPe 10/10
15	Mas-Casadesús O, de la Torre-Pérez L, Reig-Garcia G, Mas-Casadesús A, Berenguera A, Juvinyà-Canal D^(30)^	Building community engagement with caregivers through online interaction and a salutogenic approach in a period of isolation.	cualitativa con un enfoque socioconstructivista y fenomenológico, y muestreo intencional.	7 cuidadores	España (2024)	SCOPUS	CASPe 9/10
16	Alonso-Rodríguez ML, Chaparro-Díaz L, Carreño-Moreno S^(31)^	Soledad, ansiedad, depresión y adopción del rol del cuidador de personas con enfermedad crónica en San Gil, Colombia.	Estudio descriptivo, analítico de corte transversal.	115 Cuidadores	Colombia (2023)	SCOPUS	CASPe 10/10
17	Haugland BSM, Hysing M, Sivertsen B^(32)^	Study progress, recreational activities, and loneliness in young adult carers: a national student survey.	Encuesta nacional de estudiantes de educación superior en Noruega, iniciada por las tres mayores organizaciones de bienestar estudiantil [Sammen (Bergen y alrededores), Sit (Trondheim y alrededores) y SiO (Oslo y Akershus)	40 205 participantes, de 18 a 25 años.	Noruega (2022)	SCOPUS	CASPe 10/10
18	Guets W, Perrier L^(33)^	Determinants of the need for respite according to the characteristics of informal carers of elderly people at home: results from the 2015 French national survey	Encuesta representativa a nivel nacional, Capacités Aides et Resources des Seniors (CARE - ménage), recopilada en 2015, Estudios, Evaluación y Estadística (DREES).	6.201 (de 16 años o más) Los cuidadores informales	Francia (2021)	SCOPUS	CASPe 10/10
19	Lwin M, Sheldenkar A, Panchapakesan C^(34)^	A Digital Mobile Community App for Caregivers in Singapore: Predevelopment and Usability Study.	Encuesta previa al desarrollo para identificar las necesidades específicas de los cuidadores y las brechas en las redes comunitarias basadas en la web actualmente disponibles	103 cuidadores	Singapur (2021)	SCOPUS	CASPe 9/10
20	van Woerden HC, Angus N, Kiparoglou V, Atherton I, Leung J^(35)^	Long-Term Conditions in Older People are Linked with Loneliness, but a Sense of Coherence Buffers the Adverse Effects on Quality of Life: A Cross-Sectional Study.	Encuesta transversal de una muestra aleatoria de 3000 hogares en un área definida del norte de Escocia (NHS Highland).	1471 encuestas	Escocia (2021)	SCOPUS	CASPe 10/10
21	Rippon I, Víctor CR, Martyr A, Matthews FE, Quinn C, Rusted JM, et al.^(36)^	Perspectivas diádicas sobre la soledad y el aislamiento social entre personas con demencia y cuidadores conyugales: hallazgos del programa IDEAL, Aging & Mental Health	Estudio cualitativo se realizaron entrevistas semiestructuradas	a 11 personas cuidadores	England, Scotland, and Wales (2023)	PUBMED	CASPe 10/10
22	Ross A, Pérez A, Wehrlen L, Lee LJ, Yang L, Cox R, et al.^(37)^	Factors influencing loneliness in cancer caregivers: A longitudinal study	Estudio prospectivo de medidas repetidas.	129	EEUU (2020)	PUBMED	CASPe 10/10
23	Fee A, McIlfatrick S, Ryan A^(38)^	Examining the support needs of older male spousal caregivers of people with a long-term condition: A systematic review of the literature.	Búsquedas sistemáticas se aplicaron criterios de elegibilidad a los textos completos de 104 artículos y 11 artículos cumplieron con los criterios de inclusión.		Reino Unido (2020)	SCOPUS	CASPe 9/10
24	Henriques NL, Silva JB, Charepe ZB, Braga PP, Duarte ED^(39)^	Factors that promote and threaten Hope in caregivers of children with chronic conditions	Estudio cualitativo, descriptivo, exploratorio,	46 cuidadores familiares	Brasil (2023)	SCOPUS	CASPe 10/10
25	Mildrum Chana S, Álvarez L, Poe A, Bibriescas N, Wang DH, DiFiglia S, et al.^(40)^	The Daily Experiences of Hispanic and Latinx Dementia Caregivers Study: Protocol for a Fully Remote Daily Diary Observational Cohort Study.	Este estudio longitudinal a escala de tiempo múltiple	500 cuidadores hispano americanos	Hispanoamérica (2024)	PUBMED	CASPe 9/10
26	Hajek A, Kretzler B, König HH. Review^(41)^	Loneliness and Social Isolation: A Systematic Review.	Revisión sistemática	12 estudios de América del norte y Europa	América del norte y Europa (2021)	PUBMED	CASPe 9/10
27	Manskow US, Sigurdardottir S, Røe C, Andelic N, Skandsen T, Damsgård E, et al.^(42)^	Factors Affecting Caregiver Burden 1 Year After Severe Traumatic Brain Injury: A Prospective Nationwide Multicenter Study.	Estudio multicéntrico nacional prospectivo	92 Cuidadores	Noruega (2015)	SCOPUS	CASPe 9/10
28	Manrique-Anaya Y, Barrios-Puerta Z, Chaparro-Díaz L, Carreño-Moreno SP^(43)^	Adopción del rol y soledad asociado al cuidador familiar de personas con enfermedad crónica.	Estudio analítico transversal.	390 Cuidadores	Colombia (2024)	SCOPUS	CASPe 10/10
29	Ris I, Schnepp W, Mahrer Imhof R^(44)^	An integrative review on family caregivers’ involvement in care of home-dwelling elderly	Integrative review	26 Estudios	Alermania (2019)	PUBMED	CASPe 9/10
30	Vasileiou K, Barnett J, Barreto M, Vines J, Atkinson M, Lawson S, et al.^(45)^	Experiences of Loneliness Associated with Being an Informal Caregiver: A Qualitative Investigation. Front Psychol	Método mixto	16 cuidadores	Reino Unido (2017)	PUBMED	CASPe 10/10

La investigación permitió definir la estructura operacional del fenómeno de soledad en los cuidadores de personas con ECNT, su origen multidimensional, trayectoria desde la identificación de los factores de vulnerabilidad o antecedentes, atributos y consecuencias, para confirmar su complejidad como se ilustra en el Cuadro 3.

**Cuadro 3:** Estructura esencial del análisis de concepto “soledad” en los cuidadores de personas con ECNT, 2024 (N = 30)

Antecedentes	Atributos	Consecuencias
**Físicos:** edad (adulto joven/maduro), género femenino, autopercepción de mala salud, tiempo dedicado al cuidado >20 horas, nivel de dependencia del paciente^(18,21,22,33,35-37)^.	**Percepción de soledad emocional** **Físico:** cansancio, malestar físico, dolores musculares, cefalea, vivir solo^(18),19)^, viudez^(20)^	**Físico :** sensación de malestar, frustración, incomodidad, agotamiento, fatiga, vacío, estrés crónico, dolor, carga física^(18,21,23-25,27-29,32-35,38,41,42,45)^.
**Psicológicos :** relaciones interpersonales de baja calidad, pobres recursos de afrontamiento^(18,21,22,33,35-37)^, estrés del cuidador, sobrecarga, baja autoestima, personalidad pesimista^(18,25,35,37)^.	**Percepción de soledad emocional** **Psicológicos :** personalidad pesimista^(21)^, sobrecarga, estrés asociado al cuidado, ansiedad, y depresión^(22-30)^; estos sentimientos se integraron a la percepción de soledad emocional.	**Psicológico:** aflicción, depresión, ansiedad, irritabilidad, problemas de salud mental, tensión en las relaciones personales^(18,21,23-25,27-29,32-35,38,45)^ y ansiedad^(30)^.
**Sociales:** vivir solo^(18,21,22,33,35-37)^, retiro obligado del trabajo^(27)^, imposibilidad de participar en relaciones y actividades ordinarias disponibles^(30)^, bajo apoyo familiar e institucional^(3),8)^, expectativas del personal sanitario y los miembros de las familias^(19,27,28,32,33,38-44)^.	**Percepción de soledad Social** **Social:** sentirse aislados, solos, sin apoyo, malestar por pérdida de su red social^(3),27)^, bajas relaciones con familiares y amigos^(8),31)^, sentimientos de exclusión, aislamiento, sobrecarga social^(32),33)^	**Social:** interfiere en el desempeño el rol social, puede desencadenar pérdida de relaciones de amigos, familia y vida social, efectos negativos con sentimientos de frustración^(29),42)^, y soledad que impide emprender nuevos roles al tiempo^(31)^.
**Espirituales:** cansancio, desesperanza, baja autopotencia y autoeficacia, incertidumbre, emociones que acrecientan la sensación de soledad^(20,28,29,33,34,38,39)^.	**Percepción de soledad Social** **Espirituales:** dificultad para pedir ayuda y encontrar personas afines^(34)^, bajo sentido de coherencia, altos niveles de soledad social^(35)^, bajas habilidades sociales^(20)^.	**Espirituales:** incertidumbre, proyecto de vida confuso, y desesperanza^(18,21,23-29,32-35,38,39)^.

1. Selección del concepto “soledad” en los cuidadores de personas con ECNT

Analizar el concepto de soledad es significativo en la ciencia y el ejercicio de enfermería . Se justifica por su aumento creciente con la pandemia COVID-19, definido como problema de salud pública mundial, al ser un factor de riesgo para padecer enfermedades y afectación de la salud integral^(3)^.

2. Propósito del análisis del concepto “soledad” en los cuidadores de personas con ECNT.

Analizar el concepto de soledad en los cuidadores de personas con ECDS representó una exploración amplia y sistemática del concepto, fundamentada en la comprensión del fenómeno para guiar la práctica del cuidado en los cuidadores informales.

3.Identificación de los usos del concepto “soledad” en los cuidadores de personas con ECNT.

En coherencia con la literatura, el concepto es un fenómeno experimentado por todos los individuos durante su vida, susceptible de ser percibida^(8)^ por los cuidadores como soledad emocional y social.

4. características o atributos definitorios del concepto de “soledad” en cuidadores de personas con ECNT.

Los hallazgos encontrados permitieron enunciar las propiedades de la soledad percibida entérminos desufrimiento físico, emocional, social y espiritual. En las dimensiones física y emocional se incluyeron manifestaciones de cansancio, malestar físico, dolores musculares, cefalea, vivir solo^(18),19)^, viudez^(20)^, personalidad pesimista^(21)^, sobrecarga, estrés asociado al cuidado, ansiedad, y depresión^(22-30)^; estos sentimientos se integraron a la percepción de soledad emocional.

En relación al sufrimiento social y espiritual reportaron sentirse aislados, solos, sin apoyo, malestar por pérdida de su red social^(3,27)^, bajas relaciones con familiares y amigos^(8),31)^, sentimientos de exclusión, aislamiento, sobrecarga social^(32),33)^, dificultad para pedir ayuda y encontrar personas afines^(34)^, bajo sentido de coherencia, altos niveles de soledad social^(35)^, bajas habilidades sociales^(20)^, contexto articulado a la percepción de soledad social, como se presenta en el Cuadro 3.

5. Identificar los antecedentes del concepto “soledad” en los cuidadores de personas con ECNT

La soledad en los cuidadores se genera por múltiples causas, la vulnerabilidad descrita en la literatura vislumbró factores físicos, psicológicos, sociales y espirituales predictores de percepción de soledad relacionados con brindar cuidados, como lo ilustra el Cuadro 3.

Físicos: edad (adulto joven/maduro), género femenino, autopercepción de mala salud, tiempo dedicado al cuidado >20 horas, nivel de dependencia del paciente^(18,21,22,33,35-37)^.

Psicológicos: relaciones interpersonales de baja calidad, pobres recursos de afrontamiento^(18,21,22,33,35-37)^, estrés del cuidador, sobrecarga, baja autoestima, personalidad pesimista^(18,25,35,37)^.

Sociales: vivir solo^(18,21,22,33,35-37)^, retiro obligado del trabajo^(27)^, imposibilidad de participar en relaciones y actividades ordinarias disponibles^(30)^, bajo apoyo familiar e institucional^(3),8)^, expectativas del personal sanitario y los miembros de las familias^(19,27,28,32,33,38-44)^.

Espirituales: cansancio, desesperanza, baja autopotencia y autoeficacia, incertidumbre, emociones que acrecientan la sensación de soledad^(20,28,29,33,34,38,39)^.

6. Consecuencias relacionadas con el concepto “soledad” en los cuidadores de personas con ECNT.

La soledad surge como consecuencia de brindar atención en el hogar, considerada al mismo tiempo factor predictor de sufrimiento con consecuencias en el bienestar.

Físico: sensación de malestar, frustración, incomodidad, agotamiento, fatiga, vacío, estrés crónico, dolor, carga física^(18,21,23-25,27-29,32-35,38,41,42,45)^.

Psicológico: aflicción, depresión, ansiedad, irritabilidad, problemas de salud mental, tensión en las relaciones personales^(18,21,23-25,27-29,32-35,38,45)^ y ansiedad^(30)^.

Social: interfiere en el desempeño el rol social, puede desencadenar pérdida de relaciones de amigos, familia y vida social, efectos negativos con sentimientos de frustración^(29,42)^, y soledad que impide emprender nuevos roles al tiempo^(31)^.

Espiritual: incertidumbre, proyecto de vida confuso, y desesperanza^(18,21,23-29,32-35,38,39)^.

El Cuadro 3, resume la estructura del concepto soledad en los cuidadores de personas con ECNT., donde se presentan los antecedentes, atributos y consecuencias.

De acuerdo con los resultados, obtenidos del proceso de síntesis de las diferentes investigaciones, se logró reflexionar sobre la trayectoria de brindar cuidados a la persona con ECNT, desde los antecedentes, la naturaleza dinámica del cuidado en el transcurso del tiempo, y los efectos e impactos en todos los dominios de la vida de los cuidadores de personas con ECNT, proceso que aporto mayor claridad al marco contextual de este fenómeno, como se presenta en la Figura 2.

**Figura 2:** Marco contextual del análisis de concepto “soledad” en los cuidadores de personas con ECNT, 2024

7. Definir los referentes empíricos del concepto “soledad” en los cuidadores de personas con ECNT.

Este paso es fundamental para presentar la forma de medir o cuantificar los conceptos en la realidad. El síntoma más común manifestado entre los cuidadores fue la carga, pero también destacaron la pérdida de espacio personal y los sentimientos relacionados con la ansiedad y sentirse solo.

8. Caso modelo del concepto “soledad” en los cuidadores de personas con ECNT.

Se evidencian los atributos definitorios de la soledad en cuidadores:

Alicia tiene 55 años y desde hace once cuida a su esposo Jaime, quien fue diagnosticado con Alzheimer a los 52 años. Se describe como una persona trabajadora y con vocación de ayuda a los demás. Convive con Jaime y dos cuñadas, ninguno de los cuales tiene empleo. Desde que su esposo enfermó, Alicia tuvo que dejar su negocio para dedicarse por completo a su cuidado y al sostenimiento de la familia. Esta situación ha generado serias dificultades económicas. La salud de Jaime se ha deteriorado progresivamente y recientemente le diagnosticaron una enfermedad oncológica. Además, Alicia enfrenta sus propios problemas de salud, lo que agrava su situación.

Inicialmente, Alicia notó cambios sutiles en el comportamiento de su esposo, aunque desconocía la enfermedad. Percibía situaciones inusuales, como relatos de experiencias en lugares donde nunca había estado, la pérdida de objetos que luego atribuía a ella o a sus hermanas. Para entender mejor lo que ocurría, empezó a informarse a través de libros, lo que le permitió comprender la enfermedad. La fase inicial fue particularmente difícil para ella, llevándola al llanto en numerosas ocasiones. Con el avance del Alzheimer, Jaime desarrolló un tumor en la nariz, por el cual fue sometido a cuatro cirugías, hasta que le confirmaron que era maligno. En octubre del año pasado, Alicia descubrió una protuberancia en la axila de su esposo y, tras realizarse una biopsia, se confirmó otro diagnóstico de cáncer.

Actualmente, Jaime enfrenta dos enfermedades complejas, mientras que Alicia experimenta un profundo desgaste emocional. Según el médico, su esposo se encuentra en la tercera fase del Alzheimer y aún es capaz de desplazarse por sí mismo. Sin embargo, ella se siente agotada, aislada y llena de incertidumbre sobre su futuro. Con frecuencia se encierra a llorar para no ser vista por los demás y manifiesta una sensación de vacío.

Los recursos económicos son insuficientes, ya que Alicia no cuenta con pensión propia y la de su esposo no cubre todos los gastos. Aunque la familia sigue unida, la convivencia se ha vuelto difícil. Sus cuñadas, por ejemplo, muestran incomodidad ante situaciones cotidianas del deterioro de Jaime, como cuando derrama comida o tiene dificultades para controlar sus secreciones.

En cuanto a su propia salud, Alicia ha sido operada de la columna y tiene una placa en las vértebras cervicales. Además, sufre de fibromialgia y está a la espera de otro procedimiento médico. Muchas veces amanece con ganas de llorar debido al dolor constante, lo que le impide dormir bien. A pesar de esto, sigue compartiendo la cama con Jaime, pues él experimenta miedos, alucinaciones y ve insectos inexistentes, lo que le genera compasión. Sin embargo, los movimientos bruscos de su esposo durante la noche la obligan a buscar descanso en el sofá hasta la madrugada, logrando apenas unas pocas horas de sueño.

Emocionalmente, Alicia experimenta constantes altibajos. La semana anterior, por ejemplo, se sintió especialmente deprimida, evitando el contacto con sus cuñadas y prefiriendo estar sola. Cuando la casa está en calma, llora en silencio para desahogarse. No puede tomar medicación para dormir, ya que necesita estar atenta a su esposo. Se siente sola en su dolor y sin apoyo, lo que la ha llevado a aislarse. Extraña al hombre que su esposo solía ser, un compañero atento y amoroso, y le resulta profundamente doloroso verlo apagarse poco a poco.

9. Caso relacionado del concepto “soledad” en los cuidadores de personas con ECNT.

Se exponen algunos atributos del concepto de soledad: Carmenza mujer de 60 años, comenta a su hija “No decidí ser la cuidadora de la abuela, me tocó”. Ella empezó a ver deterioro y olvido en su madre, era la que estaba cerquita. Aunque Miguel otro hijo vivía a unas cuadras, él era hombre. Carmenza se pregunta ¿Y por qué no fue él el cuidador? Porque él es hombre. Porque él tenía su hogar, y sus cosas... “Esa es una pregunta verraca manifiesta”. Piensa que hay más disponibilidad en una mujer, para ser cuidadora. A ella nadie le dijo que fuera cuidadora. Fue la vida la que la llevó y ella no bajo los brazos. No tuvo opción. Ellos [sus hermanos] nunca pensaron en hacerse responsables de los padres.

Carmenza no cree que sea solamente por ser mujer, porque la hermana es mujer y a ella no le tocó. Piensa que la persona se hace cuidadora porque tiene una sensibilidad diferente a los demás, y ellos lo aprovechan. El cuidador decide está ahí, ya no puede salir. Ella piensa: “voy a hacerlo por hoy”, pero cuándo se da cuenta, está sola con toda la responsabilidad, sola, aislada, le toco dejar de trabajar y encerrarse con sus padres, ya no pudo salir de ese laberinto.

Ella dice: hay roles que supuestamente ella debe cumplir. Su núcleo familiar es más consciente, ellos son autosuficientes y así ella los puede dejar de lado un poco y poner la cara al cuidado de su madre. Ella ha tenido problemas porque antes de esta situación, ella había acordado unos roles con su esposo, y ahora deben ser cambiados, cuando ella está bien con su esposo, puede estar mejor allá con su madre.

## DISCUSIÓN

Este análisis conceptual evidencia la complejidad del fenómeno descrito, la soledad en los cuidadores es el desenlace de diversas circunstancias entrelazadas, que en algún momento las personas pueden experimentar en ciertas situaciones y condiciones especiales^(1,22,23)^. Actualmente la soledad es considerada un problema y un desafío para la salud pública por su relación con bajo bienestar y afectación de la calidad de vida en los cuidadores, quienes la perciben como una sensación desagradable que favorece estados negativos para la salud, condiciones que aumentan la presión arterial, marcadores de inflamación, ocasionan deterioro cognitivo, disfunciones orgánicas, trastornos psicofisiológicos y padecimientos crónicos, que en conjunto pueden aumentar la mortalidad^(13)^.

Es crucial diferenciar la soledad como una experiencia subjetiva de aislamiento emocional y aislamiento social como una situación objetiva de falta de contacto social. El deterioro funcional y la dependencia de las personas con enfermedades crónicas no transmisibles (ECNT), sumados al aislamiento impuesto durante la pandemia, han acentuado la percepción de soledad entre los cuidadores. A pesar de compartir el hogar con otros familiares, muchos cuidadores sienten que la responsabilidad del cuidado recae exclusivamente sobre ellos. Esta sensación se intensificó debido a la limitada presencia de profesionales de la salud en los hogares durante la crisis sanitaria^(24),30)^.

La soledad en los cuidadores es definida como la sensación de estar solos, sin alguien con quien hablar, incomprendidos, con necesidad de compañía, ser tenidos en cuenta^(25,31,32,34)^; percepción de angustia por falta de conexión social^(26)^; sentimiento de aislamiento social resultado de las demandas del cuidado^(18,27,33,35,36)^. Quienes cuidan sin apoyo familiar e institucional, experimentan la compleja situación emocional y social de soledad, en respuesta al estrés que supone el cuidado, así como el impacto financiero y emocional^(19),28)^.

La soledad es la condición psicológica resultante de la discrepancia entre las relaciones sociales existentes y las deseadas por el individuo^(20)^. Es el sentimiento percibido como dolor real definido en términos físicos como soledad emocional y de aislamiento como soledad social, que puede afectar la salud física, psicológica y la función familiar^(21),29)^. De manera particular, en el bienestar emocional de los cuidadores hombres, la soledad ocasiona impacto negativo acompañado especialmente de sensación de aislamiento^(38)^.

La Real Academia Española define la soledad como la ausencia, voluntaria o involuntaria, de compañía, y como un sentimiento de melancolía derivado de la pérdida o ausencia de alguien o algo^(46)^. Por otro lado, Peplau y Perlman explican que la soledad surge de un desbalance entre las relaciones sociales reales de un individuo y sus expectativas sobre ellas^(47)^.

Es importante diferenciar entre aislamiento social y soledad. Mientras que el aislamiento es una condición objetiva en la que una persona está físicamente sola, pero puede sentirse conectada con otros, la soledad es subjetiva y puede experimentarse incluso en presencia de otras personas. Este último estado suele ser doloroso y angustiante^(48)^.

Dentro de los conceptos más utilizados para describir la soledad de los cuidadores se encontraron: sensación de malestar emocional, dolor, vacío, sufrimiento, aflicción, congoja, desconsuelo, tristeza, desolación, molestia, incomodidad, desesperanza, indefensión, abandono, condiciones psicológicas resultantes de la discrepancia entre las relaciones sociales existentes y las deseadas del individuo^(20)^. Se identificaron dos tipos de soledad: la emocional, que ocasiona sentimiento de vacío, abandono y desolación por la ausencia de relaciones interpersonales significativas y, la social, donde se reconoce la falta de amigos, hermanos, y vecinos^(49)^.

A partir de esta revisión sobre el uso del concepto de “soledad” en los cuidadores, se puede afirmar que es un sentimiento negativo y poco deseable, percibido como doloroso y real, definido en términos físicos de soledad emocional y de aislamiento como soledad social, con efectos importantes en la salud física, psicológica y la función familiar^(21),29)^.

En el análisis de la literatura los atributos que mejor explican el concepto de soledad en los cuidadores se sintetizan en dos dimensiones: soledad emocional y soledad social.

La soledad emocional se define como sensación de cansancio, malestar físico, dolores musculares, cefalea, lumbalgia, y encontrarse solo^(18)^, a veces resultado de una decisión personal^(19)^, o transiciones de la vida como la viudez, vivir solo, o en área rural distante^(20)^. Se asocia a características de personalidad pesimista sobre los demás, y sobre sí mismo^(21)^, altos niveles de sobrecarga, estrés asociado al cuidado, ansiedad, síntomas depresivos, manifestaciones de falta de tiempo para el cuidado propio y adopción básica del rol^(22-29,40)^. En un modelo integral de cuidado, la percepción de soledad es un factor psicológico, que examina la capacidad de los cuidadores para vivir bien, mide la satisfacción con la vida, calidad de vida y bienestar^(36)^.

La soledad social la perciben como sentirse aislados, solos, sin apoyo, malestar por renunciar a aspectos importantes de su vida^(30)^, baja calidad de las relaciones con otros familiares y amigos^(31)^, tener pocos amigos cercanos o con quien poder hablar. Los cuidadores presentaron sentimientos profundos de exclusión y aislamiento, especialmente los jóvenes con responsabilidades de cuidado más extensas^(32)^, que viven con la persona cuidada, y dedican al cuidado un tiempo mayor a 30 horas al mes^(33)^.

Los cuidadores enfrentan grandes dificultades para encontrar personas con experiencias similares^(34)^, lo que contribuye a un bajo sentido de coherencia y un incremento en la percepción de soledad social^(35)^. También pueden presentar habilidades sociales limitadas^(21)^, lo que dificulta aún más la posibilidad de establecer vínculos de apoyo. Desde una perspectiva temporal, la soledad puede clasificarse en^(50)^:

- Soledad crónica: cuando la persona, independientemente de su entorno, siente un aislamiento persistente durante años.
- Soledad situacional: ocurre en momentos puntuales, como el duelo tras la pérdida de un ser querido.
- Soledad transitoria: responde a eventos específicos y es temporal, como los conflictos interpersonales.

Según la literatura, los factores predictores precursores de percepción de soledad fueron: edad (adulto joven/maduro), género femenino, niveles bajos de educación, relaciones sociales de baja calidad, mala autoevaluación de la salud, disminución de la funcionalidad de la persona cuidada, múltiples acciones de cuidado realizadas, percepción de ayuda recibida, vivir solo, más de 20 horas de cuidado no remunerado^(18,21,22,33,34)^.

El alto nivel de demanda en el rol de cuidador, combinado con el aislamiento social y la falta de apoyo, puede generar alteraciones en la relación con la persona cuidada^(42-45)^. Además, la reducción en la participación en redes de apoyo contribuye al estrés, la sobrecarga y el deterioro del bienestar emocional^(19,26,36,37)^. En muchos casos, esta situación lleva al abandono del empleo remunerado y puede provocar sentimientos de encierro y desesperanza^(27)^, pobres recursos de afrontamiento^(22-25,31)^, lo que se traduce en la imposibilidad de participar en relaciones y actividades ordinarias disponibles^(30)^.

La falta de apoyo familiar e institucional, las expectativas del equipo de salud y de los familiares supone una fuente de estrés y de cambios en el rol social. En este contexto aumentan los sentimientos de desesperanza, junto con las dudas sobre su autopotencia, autoeficacia, e incertidumbre frente al futuro, así se acrecienta la sensación de soledad, necesidad significativa de respiro^(20,28,29,33,34,38,39)^, con incapacidad de “resolver” los problemas diarios que enfrenta la persona enferma, impotencia, relaciones interpersonales conflictivas y un sentido de responsabilidad exclusiva que aumentan la frustración y el sufrimiento^(51)^.

Se suscitan como consecuencias relacionadas con la “soledad” efectos negativos, sentimientos de frustración^(30)^, e imposibilidad de emprender nuevos roles al tiempo^(31)^.

Asimismo, se evidenció efectos perjudiciales en la salud y bienestar del cuidador con manifestaciones de: congoja, desconsuelo, tristeza, desolación, molestia, sensación de malestar, frustración, incomodidad, agotamiento, fatiga, vacío, estrés crónico, dolor, carga física, aflicción, depresión, ansiedad, irritabilidad, problemas de salud mental, incertidumbre por falta de claridad en el proyecto de vida, desesperanza, tensión en las relaciones personales^(18,21,23-25,27-29,32-35,38)^, sufrimiento físico y emocional, mayor morbilidad por hipertensión arterial y diabetes mellitus, aumento de marcadores inflamatorios y mortalidad^(26)^.

Las expectativas de los profesionales de salud y los familiares^(19)^, pueden añadir una carga adicional, generando en algunos cuidadores la sensación de no ser capaces de continuar con su labor^(23)^. Además, el impacto de la carga del cuidado varía según el género. En el caso de los hombres, asumir este rol puede entrar en conflicto con su identidad masculina, debido a la percepción tradicional del cuidado como una tarea femenina^(38)^.

Los cuidadores suelen manifestar síntomas como estrés, pérdida de espacio personal y ansiedad, además de una profunda sensación de soledad^(30)^, y sentirse solos. Una de las escalas más utilizadas a nivel mundial como medida de la soledad es la Escala de soledad University of California at Los Ángeles (UCLA) ^(19,20,22-26,31,32)^, empleada por su estructura unidimensional, en la versión validada en español, y sometida a prueba en la población adulta^(51)^, puntuaciones bajas indican mayor soledad. La escala mide la percepción de soledad en nivel bajo, moderado y grave^(51)^.

Otra herramienta ampliamente utilizada es La Escala de Soledad de De Jong Gierveld (De Jong Gierveld & Van Tilburg, 1999)^(21,29,35,36)^, aplicada en diversos estudios a nivel internacional.

El referente empírico índice compuesto del estado de salud captura la vulnerabilidad del cuidador (HSCIi)^(33)^, ha sido utilizado para valorar la probabilidad de experimentar soledad, incluye preguntas sobre datos demográficos, problemas de salud del receptor de cuidado, problemas de salud mental y física del cuidador, uso de medios digitales, búsqueda de información y apoyo, para identificar problemas relacionados con el cuidado, apoyo brindado y lo que los cuidadores desearían de una aplicación móvil para el cuidado^(34)^, un índice de coherencia alto se relaciona con menor probabilidades de experimentar soledad.

### Limitaciones del estudio

La principal limitación de este análisis de concepto está relacionada el período de tiempo de la búsqueda teniendo en cuenta que diariamente aparecen nuevas publicaciones en las diferentes bases de datos, la ubicación geográfica de las investigaciones que hacen que sean de difícil acceso y la restricción para acceder a los artículos a texto completo en las bases de datos. Los autores controlaron estas limitaciones mediante la búsqueda en un período de tiempo de 10 años, se emplearon bases de datos con acceso a artículos completos, diferentes idiomas y regiones geográficas. Los posibles riesgos y sesgos se controlaron al eliminar la literatura gris, artículos de acceso restringido, además, los artículos fueron evaluados por los autores de manera independiente (CPCM, MVOL, VMS, RGB, JFVC, y CARP), Aunque existe consenso en la literatura sobre la percepción de la soledad en los cuidadores, ha sido estudiado desde varias disciplinas que incorporan una extensa colección de productos científicos, y al integrar entre ellos los antecedentes, atributos y consecuencias del concepto en estudio, se afinan en una expresión más clara.

Los hallazgos en este estudio permiten comprender la soledad en los cuidadores como fenómeno complejo y multidimensional, resultado de la interacción entre demandas de cuidado, condiciones personales, dinámicas familiares, y la insuficiencia de redes de apoyo^(18-36)^. Estos aspectos son coherentes con investigaciones previas que documentan la vulnerabilidad del cuidador^(18-22,27-29,32,33,35-44)^ y enfatizan la necesidad de intervenciones integrales. El análisis de la literatura evidenció, además, que la soledad impacta de manera transversal todas las dimensiones del bienestar, lo que repercute en la capacidad del cuidador para sostener procesos de acompañamiento prolongado.

En cuanto a la regionalidad de la producción científica, se identificó una concentración en Europa, con predominio del Reino Unido y en menor medida, de Noruega, Alemania, España, Francia, Países Bajos y Turquía, lo que confirma la consolidada trayectoria investigativa en esta región. En América, los aportes provinieron de Colombia, Estados Unidos, México, Cuba, Chile y Brasil, lo que evidencia la participación tanto de Norteamérica como de Latinoamérica y posiciona a Colombia como un referente regional. En Asia, los estudios se localizaron en Irán, China y Singapur, con una distribución más dispersa, pero de relevancia. En contraste, la ausencia de publicaciones en África y Oceanía revela vacíos en el abordaje global del fenómeno y plantea la necesidad de ampliar la investigación hacia estas regiones para favorecer una comprensión más amplia y diversa.

### Contribuciones a la enfermería, la salud o a las políticas públicas

Esta investigación es de fundamental importancia para la disciplina de enfermería, permite la comprensión de un fenómeno de interés para el cuidado de las personas en situación de cronicidad y sus cuidadores, su abordaje permite realizar un acercamiento al concepto de soledad en los cuidadores. A través de los hallazgos del estudio se revelan múltiples características que guían la forma en que el fenómeno de estudio se puede modificar con la planificación e implementación de políticas públicas e intervenciones fundamentadas y probadas científicamente, para prevenir la soledad, y/o minimizarla si ya está presente para así contribuir en el logro de un mejor bienestar físico, psicológico social y espiritual.

## CONCLUSIONES

Este estudio reveló la complejidad del concepto “soledad” en los cuidadores de personas con ECNT, fenómeno universalmente experimentado y percibido de manera personal y subjetiva. El abordaje del tema a partir del análisis conceptual permitió la identificación de antecedentes de vulnerabilidad y complejidad de las condiciones de brindar cuidado, caracterización de los atributos e indicó las consecuencias en el cuidador. La revisión de la literatura presentó evidencia que guía la forma en que el fenómeno estudiado se puede evitar y/o modificar con base en el razonamiento clínico frente a las necesidades reales de los cuidadores de personas con ECNT, permite proponer políticas e implementar el desarrollo de planes terapéuticos enfocados en la prevención y disminución de la soledad en los cuidadores de personas con ECNT.

## Data Availability

Los datos de la investigación están disponibles en el cuerpo del artículo.
